# Versatile GCH Control Software for Correction of Loads Applied to Forearm Crutches During Gait Recovery Through Technological Feedback: Development and Implementation Study

**DOI:** 10.2196/27602

**Published:** 2021-09-22

**Authors:** Gema Chamorro-Moriana, Jose Luis Sevillano, V Perez-Cabezas

**Affiliations:** 1 Department of Physiotherapy Area of Physiotherapy Research Group CTS-305 University of Seville Seville Spain; 2 Department of Architecture and Technology of Computers Robotics and Technology of Computers Research Group TEP-108 University of Seville Seville Spain; 3 Department of Nursing and Physiotherapy Empowering Health by Physical Activity, Exercise and Nutrition Research Group CTS-1038 University of Cadiz Cadiz Spain

**Keywords:** control and monitoring software, feedback technology, motor control, gait, crutches, assisted gait for partial weight-bearing, functional recovery of the gait, unloading of lower limb musculoskeletal injury, rehabilitation, physical therapy, lower limb, injury, injuries, feedback technology, crutches

## Abstract

**Background:**

Measuring weight bearing is an essential aspect of clinical care for lower limb injuries such as sprains or meniscopathy surgeries. This care often involves the use of forearm crutches for partial loads progressing to full loads. Therefore, feasible methods of load monitoring for daily clinical use are needed.

**Objective:**

The main objective of this study was to design an innovative multifunctional desktop load-measuring software that complements GCH System 2.0–instrumented forearm crutches and monitors the applied loads, displaying real-time graphical and numerical information, and enabling the correction of inaccuracies through feedback technology during assisted gait. The secondary objective was to perform a preliminary implementation trial.

**Methods:**

The software was designed for indoor use (clinics/laboratories). This software translates the crutch sensor signal in millivolts into force units, records and analyzes data (10-80 Hz), and provides real-time effective curves of the loads exerted on crutches. It covers numerous types of extrinsic feedback, including visual, acoustic (verbal/beeps), concurrent, terminal, and descriptive feedback, and includes a clinical and research use database. An observational descriptive pilot study was performed with 10 healthy subjects experienced in bilateral assisted gait. The Wilcoxon matched-pairs signed-rank test was used to evaluate the load accuracy evolution of each subject (ie, changes in the loads exerted on crutches for each support) among various walks, which was interpreted at the 95% confidence level.

**Results:**

GCH Control Software was developed as a multifunctional desktop tool complementing GCH System 2.0–instrumented forearm crutches. The pilot implementation of the feedback mechanism observed 96/100 load errors at baseline (walk 0, no feedback) with 7/10 subjects exhibiting crutch overloading. Errors ranged from 61.09% to 203.98%, demonstrating heterogeneity. The double-bar feedback found 54/100 errors in walk 1, 28/100 in walk 2, and 14/100 in walk 3. The first walk with double-bar feedback (walk 1) began with errors similar to the baseline walk, generally followed by attempts at correction. The Wilcoxon matched-pairs signed-rank test used to evaluate each subject’s progress showed that all participants steadily improved the accuracy of the loads applied to the crutches. In particular, Subject 9 required extra feedback with two single-bar walks to focus on the total load. The participants also corrected the load balance between crutches and fluency errors. Three subjects made one error of load balance and one subject made six fluctuation errors during the three double-bar walks. The latter subject performed additional feedback with two balance-bar walks to focus on the load balance.

**Conclusions:**

GCH Control Software proved to be useful for monitoring the loads exerted on forearm crutches, providing a variety of feedback for correcting load accuracy, load balance between crutches, and fluency. The findings of the complementary implementation were satisfactory, although clinical trials with larger samples are needed to assess the efficacy of the different feedback mechanisms and to select the best alternatives in each case.

## Introduction

Gait is a basic motor function of humans [1-3]; thus, many health (eg, physiotherapy, orthopedics, biomechanics) and engineering (eg, computing, mechanics, robotics) professionals, among others, have pooled their efforts to analyze and assess gait for recovery or optimization purposes.

New feedback-based technologies are especially useful in the field of rehabilitation [4] to reeducate an altered function or teach a new one, such as in the functional recovery of walking [5,6]. These aspects are fundamental objectives of physiotherapy [7,8], which enable subjects to voluntarily control and modify certain body functions or biological processes if they are given new information about these functions and processes. This is the basic principle underlying feedback mechanisms [7,9,10].

This paper focuses on *extrinsic feedback*, which is provided by external sources [11,12]. The wide range of well-known feedback technologies include visual [5,13-15], acoustic [16-18], and haptic [11,16,19] technologies, which are usually adapted to each individual user in a coherent manner [20]. Other classifications and types of feedback depend on when the information is applied, what type of data it offers, or what objective it pursues, among other factors. Some confusing terms in the literature are clarified below.

First, when feedback is given, it can be *concurrent* (ie, *simultaneously* provided during the intervention) or *terminal* (ie, retarded or postresponse feedback, provided when the action is finished). Concurrent feedback can be *continuous* or *intermittent*. Terminal feedback can be *immediate* [21], *delayed,* or even *summary* (provided after several repetitions of the movements or actions) [22].

Second, according to the type of information received, the feedback can be classified as *knowledge of performance* (KP) [23], which offers performance features (eg, if the subject keeps looking ahead while walking) and *knowledge of results* (KR) [24,25], which involves judgment of a correct or wrong action, or calculation of scores using rating scales. Feedback can also be *descriptive* or *prescriptive*, providing information about how to correct the errors [22]. For example, a physical therapist may describe a gait error by saying that patients are looking at their feet, or will try to correct the error by explaining that they should look at a fixed point ahead of them because the ground is smooth and they do not need to look down.

The extraordinary diversity of available feedback and the creativity in how it is applied have increased the opportunities for adapting and optimizing each intervention based on the user’s needs.

Technological advances have prompted the development of new retraining walking techniques based on feedback provided by monitored instrumentation systems and associated software, as confirmed in various studies [14,15,26,27]. In general, these instruments capture real-time information on the gait [2,4,6,13,15-18,20,21,27], allowing the clinician to decide whether the user should receive concurrent feedback or whether it is more convenient to delay the information and verbally provide terminal feedback.

Functional recovery of walking and assisted gait are generally related to different areas of health, including neurology [28,29], gerontology [30-32], and rheumatology [33]; however, one context in which the need to control and optimize the loads applied to the forearm crutches is most clearly evident involves patients with a lower limb musculoskeletal injury [34], who have to relearn how to automate the walking gesture correctly [35]. Traumatological physiotherapy therefore very often involves using forearm crutches to partially unload the injured lower limb, progressing to full weight-bearing. Examples of such applications include sprained knees [36] or ankles [37], surgery for knee meniscopathies [38], and hip arthroplasties [21]. Consequently, the load exerted on the body part in question is an essential assisted gait parameter [39], and therefore this load must be objectified and controlled to optimize it in line with the pathology, recovery process phase, and user's characteristics. Another factor is the current tendency to strongly recommend that the affected lower limb support as much weight as possible without damaging the injury. Underloading could lead to circulatory and muscle tone deficits, resulting in a decrease in osteoblastic action and increase in osteoclastic action, as well as inhibition of the joint and muscle plantar proprioceptive receptors that would imply a functional deficit. In contrast, overloading could lead to compressions or undue stress of structures even without regeneration or in the recovery process [6].

Load-measuring tools are required to both determine the ideal load in each case, which entails specific action protocols based on scientific evidence, and to objectively and progressively increase the loads [4]. At present, there are some instruments available that measure loads, such as insoles [4] or force platforms [2,15]; however, these tools do not allow for monitoring the loads exerted on the forearm crutches in every type of support, the balance between them (in bilateral-assisted gait), the simultaneity of their supports, or even their rhythm, among other relevant factors. These drawbacks are overcome by the use of instrumented forearm crutches such as the GCH System [6] that can monitor the loads applied to them so that the loads applied to the structure in question are reduced. However, these tools must be used together with a program that allows users and/or health professionals, in clinical or laboratory conditions, to obtain as much information as possible about the assisted gait performance. To the best of our knowledge, there is no scientific evidence related to the control software of instrumented forearm crutches and its implemented feedback mechanisms. As a result, the main objective of this study was to design an innovative multifunctional desktop load-measuring software that complements the GCH System 2.0–instrumented forearm crutches and monitors the loads applied to them, displaying real-time graphical and numerical information, as well as the correction of inaccuracies through feedback technology during assisted gait. The secondary objective was to perform a preliminary implementation study of the newly developed software.

## Methods

### GCH System 2.0

GCH Control Software implements GCH System to measure the loads applied to forearm crutches. Its application is particularly suitable in physiotherapy and traumatology, fields that frequently work on the functional recovery of gait for patients with lower limb musculoskeletal injury [40,41]. The rehabilitation process starts with total or partial unloading of the injured limb, followed by a gradual increase in loads aided by crutches until achieving complete functional recovery [42].

The instrumented crutches must first be described to understand how the software works. GCH System involves the coupling of a miniature force sensor inside the distal area of the crutch, and the measures are wirelessly transmitted. Each crutch contains an ultralow-power microcontroller with an input voltage of 2.4 V direct current and battery/autonomy of 6000 mAh (8 hours). The outgoing signal is detected by a USB receiver connected to a computer (“fixed system”) using a virtual communication port, or by a receptor built into a watch, mobile phone, pendant, or other portable device. In the latter case, patients have autonomy to practice aided gait outdoors with the usual obstacles encountered in daily life, such as steps and slopes, without any professional supervision. The technical specifications of GCH System 2.0 are reported elsewhere [6]. Each of these fixed and portable systems requires an independent implementation program with different features to allow each patient to achieve their specific therapeutic goals. The fixed system is described below in line with the main objective of the study.

### GCH Control Software 1.0

#### Design

GCH Control Software is an instrumentation program that has to be used on a PC; therefore, it was designed for use during patient care in the clinic or in a laboratory for research purposes.

GCH Control Software was programmed in C# in a Windows operating environment, and another program is needed as a server (GCH Server). Several GCH Control Software sessions can run simultaneously on the same server so that several patients can use the instrumented crutches at the same time. Other specific programs such as Framework 4.0 by Microsoft’s SqLite libraries for Windows and the Codejock graphical software libraries may have to be installed.

The software was developed by the first author (GC) and is registered in the Andalusia Regional Intellectual Property Registry under file number SE-690-15 and registration number 201599901533465. The source code is not available. Note that the Spanish language is used in all messages as the software was designed for use with Spanish patients.

The software translates the crutch sensor signal in millivolts into units of force (kilogram-force, which is a nonstandard unit but commonly used in our clinical context), records and analyzes the data, and provides a real-time on-screen numerical and graphical display of the loads exerted by the subject on one crutch if the assisted gait is unilateral or on two crutches if it is bilateral. The program records data up to 80 Hz, providing effective curves of weight-bearing at each crutch support.

**Figure 1:**
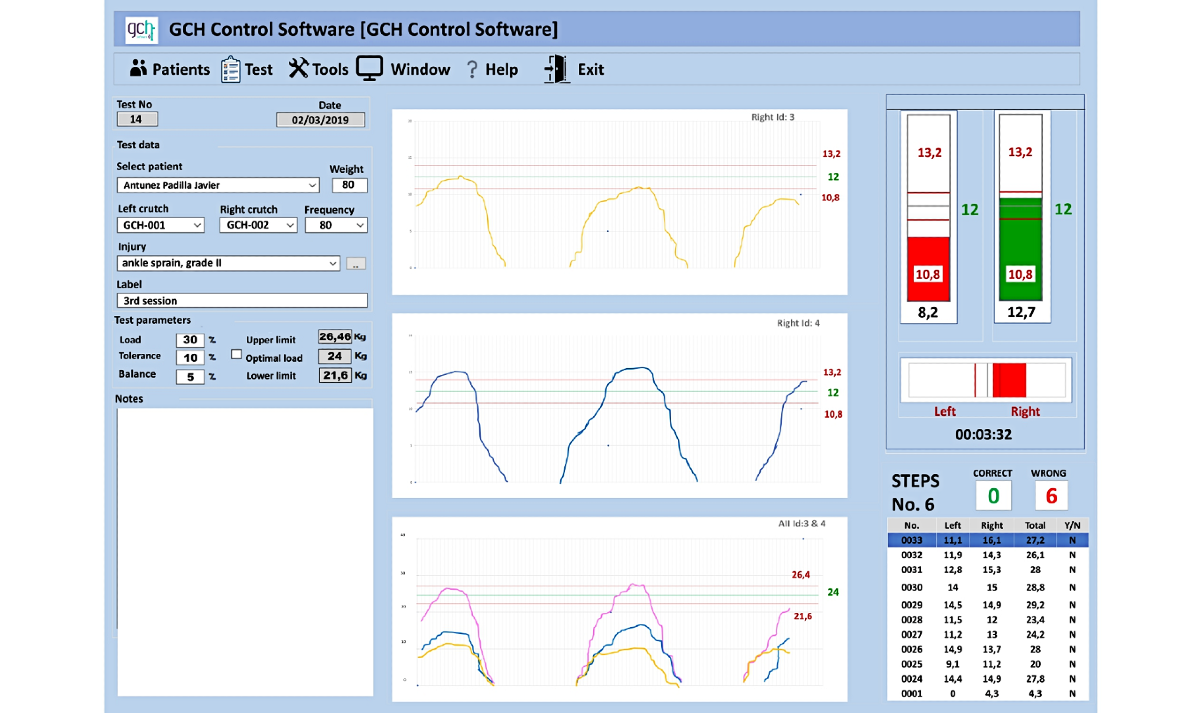
Program interface screenshot. From top to bottom: the left column shows user’s data, clinical test data, and observations; the middle shows line graphs of right crutch load, left crutch load, right and left crutch overlapping graphs, and the sum of both loads; and the right column shows double bar (right and left crutch vertical bars), balance bar, and correct and wrong crutch step counter (according to the load accuracy).

Figure 1 shows the general interface of the program, displaying data about patients, pathologies, crutch loads recommended by the physiotherapist, permitted plus or minus tolerance margins of error in the maximum loads of each crutch step and in the load balance between crutches, and a notepad. The line graphs display the independent loads of each crutch, unified load of both crutches, and overlapping of the three previous loads. Bar graphs display the following loads: independent loads for each crutch (double bar), with the option to view the unified load (single bar), and a horizontal bar that represents the load balance between both crutches (balance bar). All of these data are displayed numerically, with a correct and wrong crutch step count (according to the load accuracy).

A detailed step-by-step description and operation of the software is provided after identifying the crutches to be used in the GCH Server.

#### Data Logging Database

**Figure 2:**
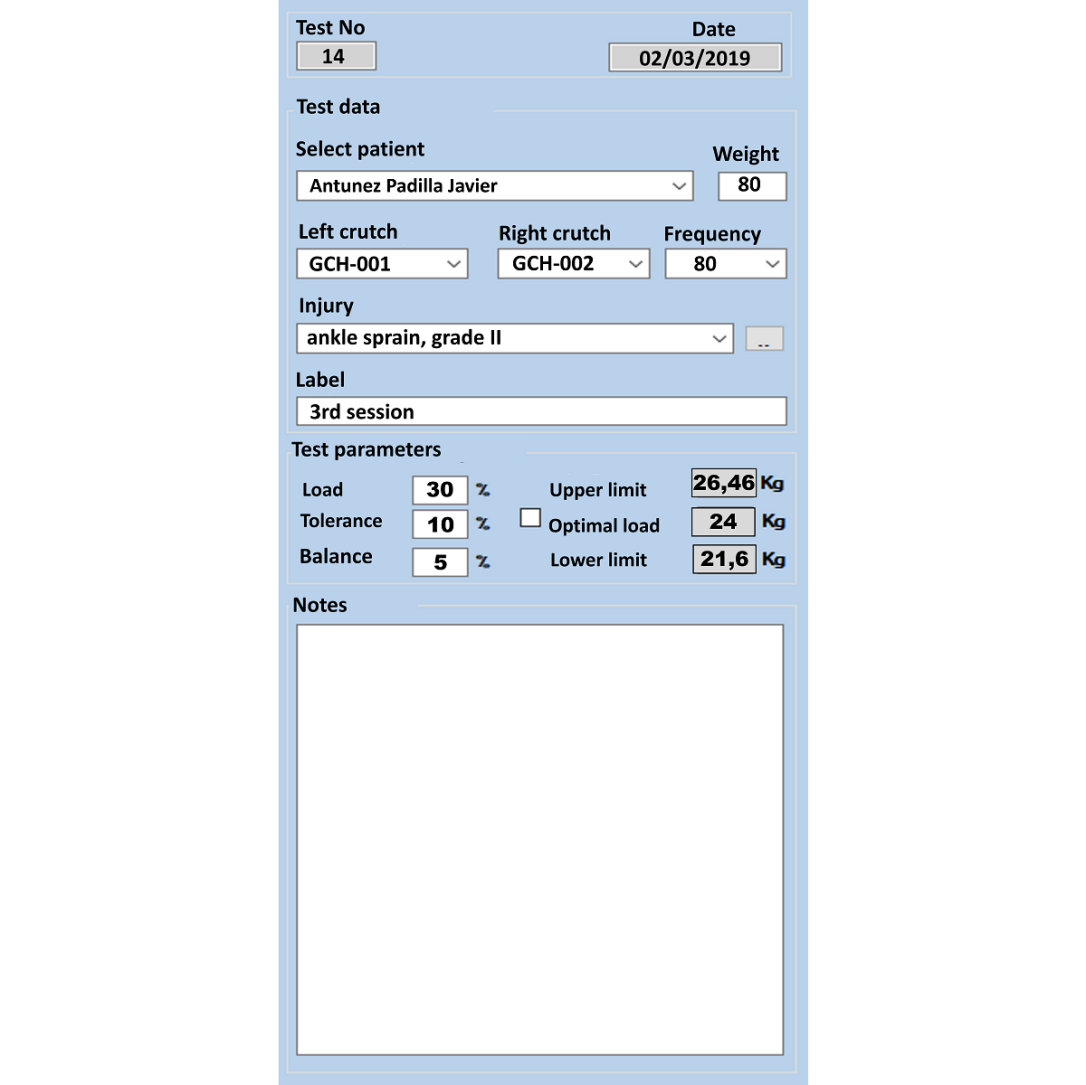
Left-hand column of the general interface shown in Figure 1, including (from top to bottom): autonumber test, date, patient’s name, current weight, crutch identification, data frequency, lesion, test label, test parameters (ideal load requested, tolerance for load accuracy errors, tolerance for imbalance between both crutches), and notepad.

The database includes user registration information such as the patient’s affiliation data and clinically relevant personal background. Each crutch is identified as “right” or “left,” and the gait is classified as unilateral or bilateral. Preoperative clinical and technical data (Figure 2) are required for an autonumber test, such as the subject’s current weight (because body weight percentages will be used), the frequency at which the data will be collected (20, 40, or 80 Hz), the current pathology, and a possible test identification label.

The next step is to record the ideal load to be exerted on the crutch, as recommended by the physiotherapist (ie, the load reduction applied to the injured limb, in percentage body weight or kilograms, which changes automatically); the permitted margins of error for overloading or underloading; and the permitted tolerance in the load balance between the two crutches, only in the case of bilateral assisted walking.

#### Graphical and Numerical Data Provided by the Software

The program uses several graphics to display the user’s walking performance in real time: line graphs (see Figure 1, middle column) and bar graphs (see Figure 1, right column).

Line graphs are only for use by the professional, in which the right crutch load, the left crutch load, and their sum are shown in different colors. There are three possible versions of line graphs: “L+R” (left + right), which shows the crutches separately; “L and R” (left and right), where a graphic overlay of the crutches is added to display both of them simultaneously; and “ALL,” which displays the superimposed graph of the two crutches plus the total load graph.

Both the researcher and clinician can view bar graphs on the computer (see Figure 1, right column, upper)*,* and the user can see these graphs displayed on an extendable screen by a projector. In this case, there are three main display options. The first is a double-bar display, which involves two vertical bars (one for each crutch), showing the different loads. This method is useful for monitoring the load amount and how it is distributed during bilateral walking to ensure it is balanced and simultaneous, without fluctuations. This version offers the most complete information. The second option is a single bar that adds up the total loads exerted by both crutches. In this case, patients focus their attention on the total load, provided that they do not have any load asymmetry problems between crutches. The information is interchangeable with the double-bar feedback display. The third display option is the balance bar. With this horizontal bar, the user’s attention focuses on balancing loads between both crutches; therefore, this display is not used in unilateral assisted gait.

All of the line and bar graphics have three horizontal lines (see Figure 1): the middle green line shows the ideal load, and the red lines above and below indicate the permitted plus or minus tolerance margin (numerical data already included in the software).

The program interface displays other interesting data such as the number of correct and wrong crutch steps in each test, along with the respective load of each crutch (see Multimedia Appendix 1).

**Figure 3:**
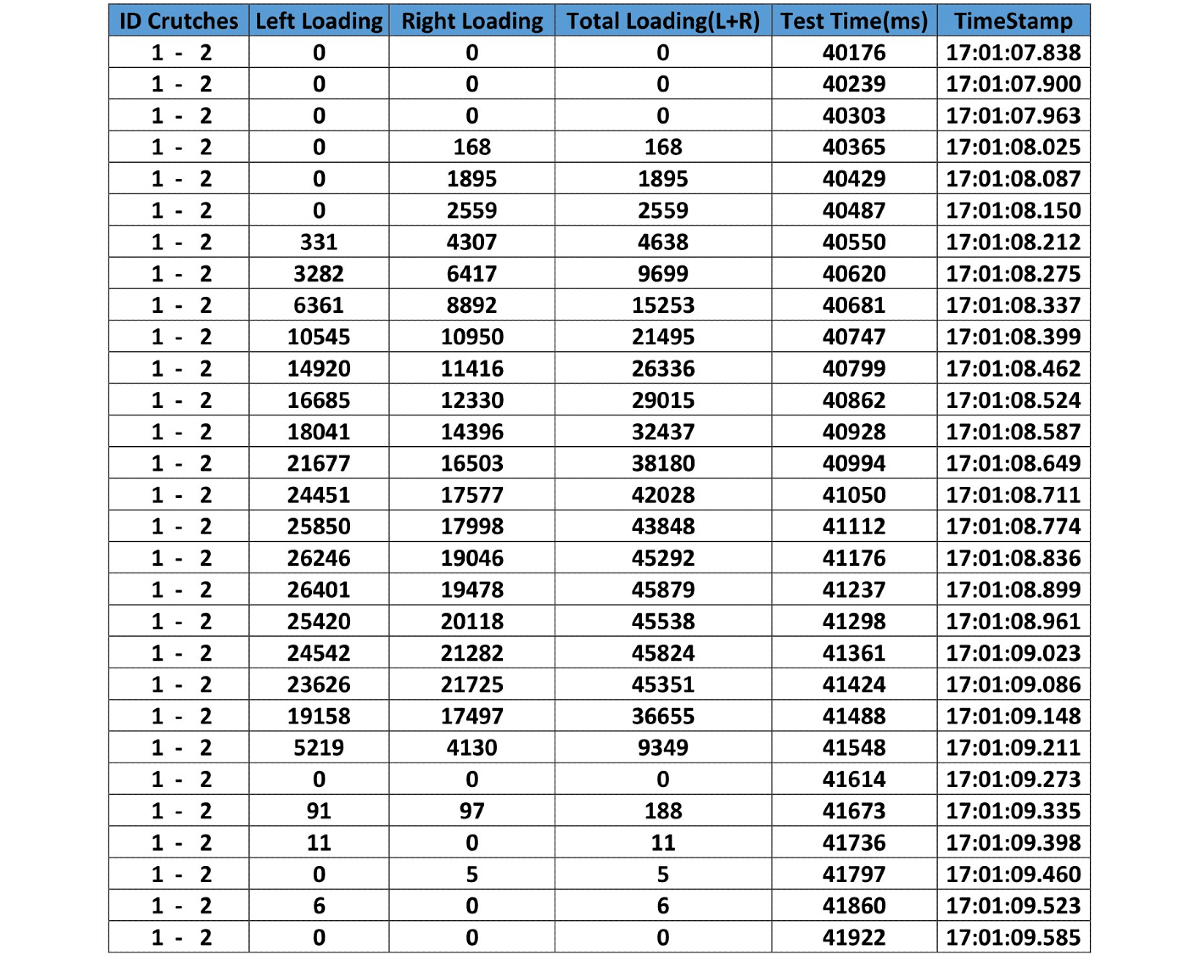
Screenshot of an example database with load results (Hz), displaying crutch identifiers, left crutch load, right crutch load, total load, time (ms), and time stamp. In this case, the right crutch starts the load earlier than the left crutch. The maximum peak of strength of the left crutch is greater than that of the right, but both coincide in time. Finally, the support time of the crutches is higher than 1 second.

**Figure 4:**
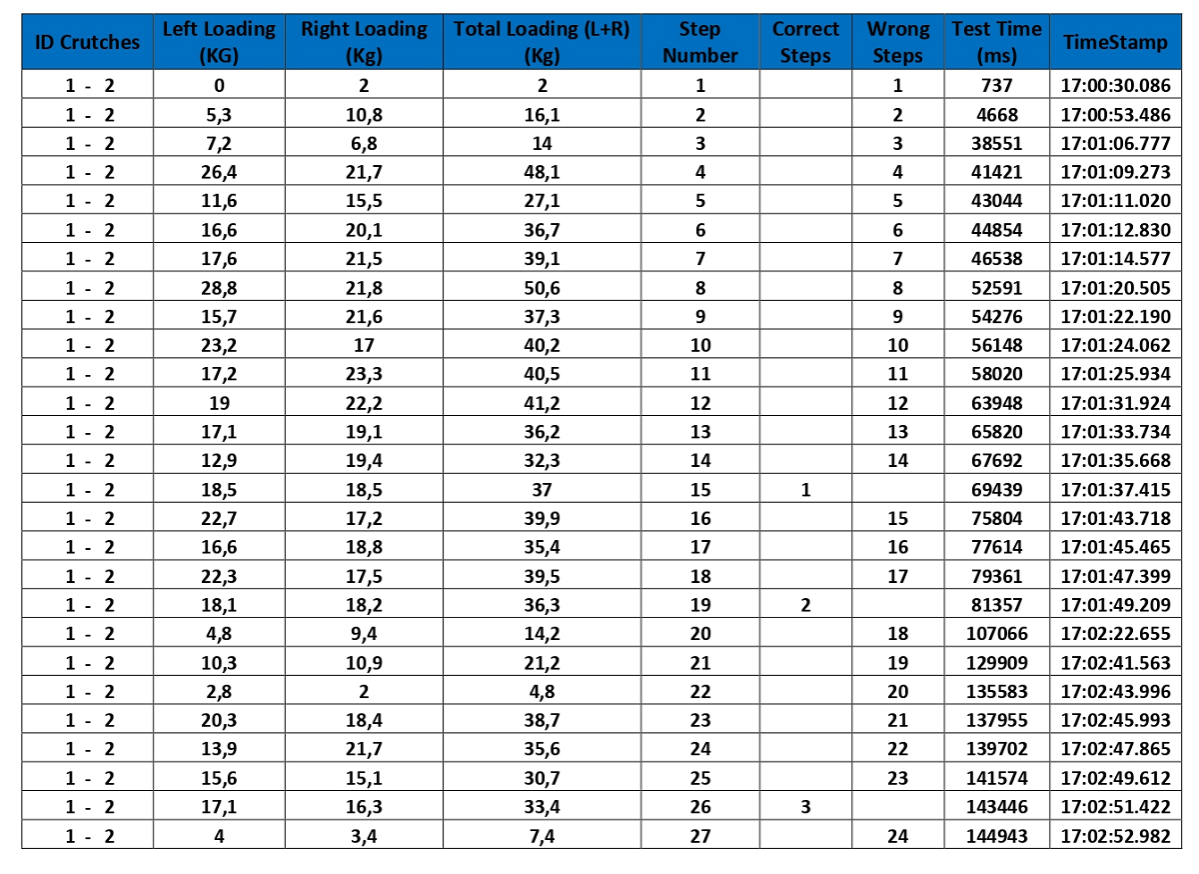
Screenshot of an example database showing maximum loads for each crutch support and feedback results, displaying crutch identifiers, maximum left crutch load, maximum right crutch load, total load, crutch support number, correct crutch supports, wrong crutch supports, time (ms), and time stamp. Note that this information enables easy assessment of loading errors, as well as of the homogeneity and symmetry between the maximum loads on the crutches.

Each test is recorded and automatically dated in the database, making it possible to access, display, and analyze graphs and numerical data regarding load accuracy, mistakes made, patient progression, and other factors, thereby enabling comparisons and statistical analyses (Figures 3 and 4).

#### Feedback Mechanism

GCH Control Software gives professionals numerical and graphic information about the loads exerted on the crutches, the precision and homogeneity of these loads, and the simultaneity or balance between crutches. All of these data can be used to assess the subjects’ assisted gait and give them oral feedback for correcting their mistakes. The software also includes a useful and feasible feedback mechanism that tells patients directly about their performance and warns them if they make mistakes while walking, which they can then self-correct. For design of the software, the most frequent and efficient feedback methods described in the literature were identified (ie, immediate visual feedback followed by terminal and immediate acoustic feedback [43]), and the most appropriate approaches were selected and implemented in the system’s user interface.

We implemented a simple beep as auditory feedback. A high-pitched beep is used when the subject overloads the affected limb, and the most annoying sound is used in this case owing to the clinical risk involved. The beep length must be short enough (ie, 0.3 seconds) to start just after the stand phase (crutch support and foot) ends and before the crutches and the foot next touch the ground. Different alternatives such as line charts, gauge charts, and bar charts were tested. Among them, vertical bar charts were found to be easier to interpret, especially if there are two bars (one for each crutch), which also makes it possible to compare the load amount and simultaneity between the two crutches.

**Figure 5:**
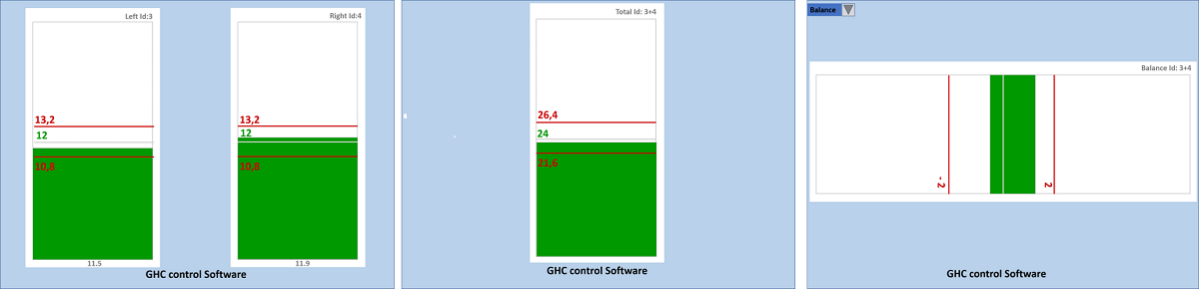
Screenshots of the three possible extendable screens projected in front of patients to show them visual feedback of the loads exerted on the forearm crutches during bilateral assisted walking. From left to right: double bar (one for each crutch), single bar (sum of both crutches), and balance bar (balance between the two crutches). The bars are green in this case because the loads are within the tolerated margins of error. The balance bar shows that the subject is applying more force to the right crutch than to the left crutch, but within the permitted margin of error.

The bar graphs mentioned above are preferably displayed to patients on an extendable screen (Figure 5), using a projector that is large enough to show straight line paths at least 16 meters long (see Clinical Implementation section). The bar graphs are applied one at a time, as each graph is designed to focus the user’s attention on the parameter that the physiotherapist considers to be the most necessary. Thus, priority is given to correcting the most serious mistakes first and the least significant mistakes last, until the assisted gait is improved, enabling the subject’s functional recovery.

Similar to the graphics that professionals see on the computer, all of the bars feature a green line to show the ideal load recommended by the physiotherapist and two red lines to indicate the minimum required or maximum allowed load (Figure 5). In the first two double-bar and single-bar versions, the bar content turns red when it is outside the tolerated load range or turns green if it is in the correct range. The error beep is therefore only heard when the red color appears to complement the visual feedback.

If a balance bar is displayed, as long as the right and left crutch load balance is maintained, the bar content stays green in the middle or slightly shifts to the side bearing the most weight (Figure 5), but without exceeding the red lines that show the error tolerance margins. If the margins are exceeded, the bar content turns red and the subject hears a beep. It does not make any sense to use this screen if the assisted gait is one-sided (ie, with only one crutch).

### Clinical Implementation

#### Study Design

Once the desktop software had been designed and developed, and the numerical and graphic measurements had been confirmed in a validation and reliability study [6], a pilot observational descriptive study was performed to obtain preliminary information on the clinical performance of the feedback mechanism implemented by GCH Control Software.

#### Participants

The sample consisted of 10 participants, including 5 women (50%) and 5 men (50%), with an age range from 21 to 55 years (mean 37.40 years, SD 11.86) and weight range between 52.6 and 81.7 kilograms (mean 64.21 kilograms, SD 9.90), all selected by nonprobability and convenience sampling. Details are given in Multimedia Appendix 2. All subjects had a normal BMI according to the US National Heart, Lung, and Blood Institute.

Inclusion criteria were healthy subjects aged between 18 and 60 years; had previous experience in partially unloading an affected lower limb by bilateral assisted gait with forearm crutches; scoring 3-4 in each of the Chamorro Assisted Gait Scale (CHAGS) items [1] (10 items, 0-4 points each); with normal gait, and asymptomatic when walking at a free cadence; and passing a simple static equilibrium test, consisting of keeping one’s balance on each foot for 30 seconds without moving the body [44].

Exclusion criteria were suffering from an evident disorder of overall coordination and physical skill that could alter the aided gait with crutches, or suffering from visual or acoustic disorders that would prevent the individual from receiving biofeedback during the intervention.

This research protocol was approved by the Ethics Committee of Virgen Macarena University Hospital (Seville, Spain). All subjects provided written informed consent before participating in the experimental study.

#### Measurements and Data Collection

Measurements were taken with GCH System 2.0 [6], and numerical and graphic data were monitored by GCH Control Software. The maximum vertical ground reaction force on the crutches was measured immediately after each support (ie, after each stride). The monitored data were immediately logged in the software database for subsequent use. The pilot implementation was carried out under laboratory conditions.

**Figure 6:**
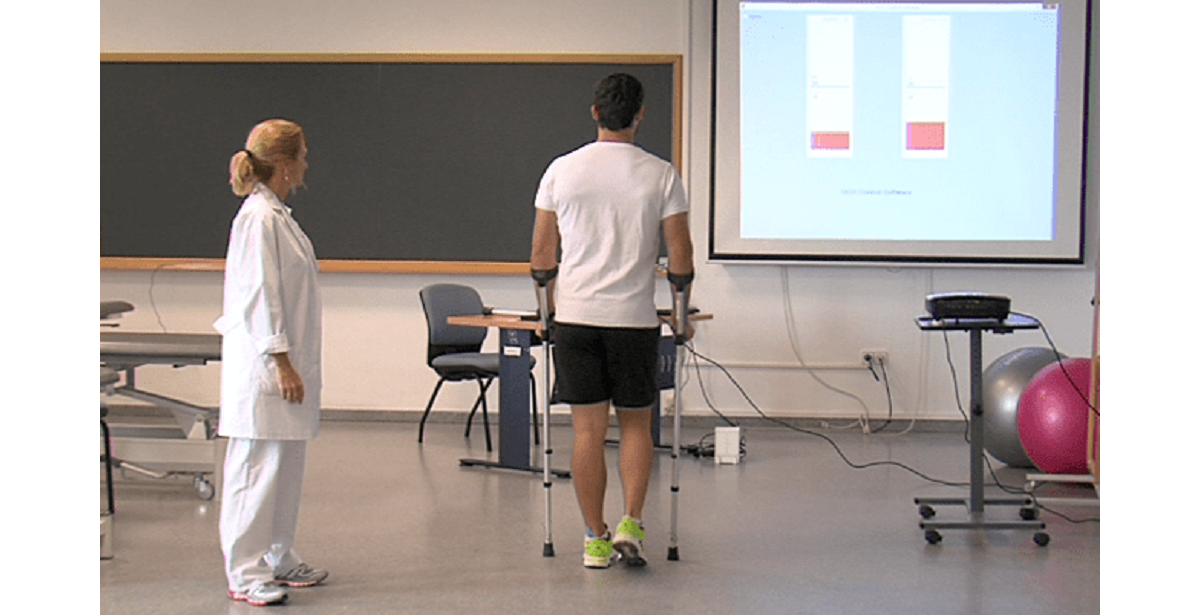
User walking toward the screen with double-bar feedback. The aided gait is bilateral (two forearm crutches) with a two-stage partial load (simultaneous heel and crutches support).

**Figure 7:**
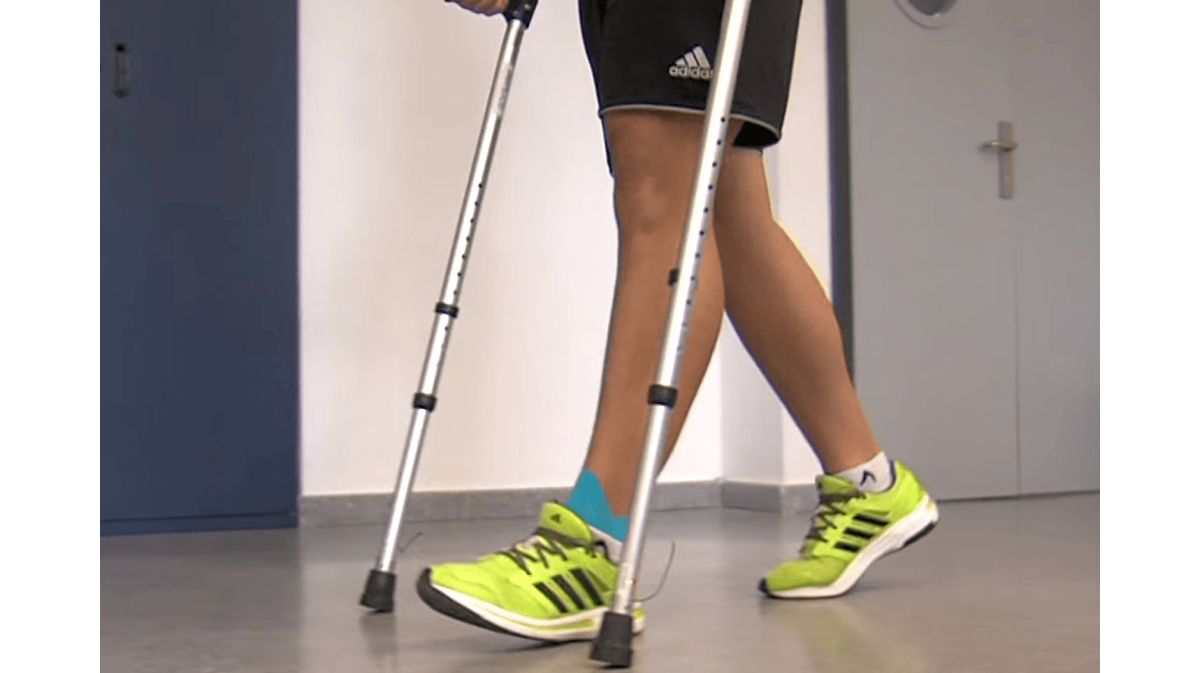
Bilateral aided gait with a partial load in two-stages, with simultaneous heel and crutches support.

The subjects walked at a free cadence, taking steps as frequently as they considered most comfortable, along 15.5 linear meters (for a minimum of 10 strides or 20 steps) on an even ground and facing a projector that displayed the image projected on the outward journey (Figure 6). They were asked to walk bilaterally with forearm crutches in two stages [1] to partially unload their previously injured lower limb. In other words, the lower limb to be unloaded is supported by the crutch (if unilateral) or by both crutches (if bilateral) at the same time. Thus, the vertical ground reaction force is applied simultaneously to the sole of the foot and to the crutches so that the subject’s body weight is distributed evenly (Figure 7).

When the subjects leaned on the injured lower limb, 50% of the body weight had to be unloaded. In other words, 50% of the ground’s vertical reaction force was applied to the crutches. A ±10% load tolerance margin was permitted (±5% of the ideal load). The crutch balance tolerance margin was 5%. For example, the two crutches should ideally bear a weight of 50 kilograms for a patient weighing 100 kilograms, but a 47.5-52.5 kilogram distribution would be considered correct. This is easy to observe in the single-bar graph (which unifies the loads). In the double-bar graph, each crutch should ideally bear 25 kilograms and a 23.75-26.25 kilogram distribution would be considered correct. Furthermore, a difference of more than 2.5 kilograms between the load of one crutch and the other is not allowed in the balance bar.

Participants were allowed to practice for 2 minutes and were reminded of the homogeneous nature of the maximum loads. Since they had mastered this type of walking, no more time was needed to confirm their automation and check that they met the correct CHAGS scale rating [1] (as described above for the inclusion criteria).

Users received visual and beep-based acoustic feedback at the same time. The physiotherapist gave no verbal feedback. The 10 participants were assessed to see if they applied the correct or wrong maximum loads requested by the physiotherapist (ie, ideal load) to the forearm crutches during bilateral assisted walking. Each participant took a minimum of four walks. They began each walk with both feet on the same line and then took their first step with the foot to be unloaded together with both crutches simultaneously. Participants performed the first walk (walk 0) without any feedback to obtain capacity baseline data, and then walked three more times with the double-bar feedback (Figure 5). That is, they were warned both visually and acoustically if they applied the wrong load to the crutches immediately at the end of each crutch support. The two independent bars also showed them when the loads applied to both crutches were simultaneous and balanced. Since this can provide too much feedback in some cases, extra walks with other simpler types of feedback (eg, three walks with a single bar or three walks with a balance bar) were applied in line with participants’ needs (up to a maximum of 10 walks). The physiotherapist decided who had to continue based on observational assessment and computer screen feedback to evaluate the tests. The system recorded 10 strides for each walk, or 10 crutch weight-bearing per stride or unloaded step. In other words, for 10 participants×10 loads (crutches)×a minimum/maximum of 4 of 10 walks, there were at least 400 measurements and a maximum of 1000 measurements available for analysis.

#### Statistical Analysis

The descriptive analysis included the means and percentages of maximum loads applied to the crutches at each support. The Wilcoxon matched-pairs signed-rank test was used to evaluate the load accuracy evolution. The analysis provided the *P* value to evaluate each subject’s progress, comparing each of their walks. The practice walks of different subjects were not compared with each other. A confidence level of 95% was considered, and the experimental *P* value was evaluated at a significance level of 5%. The data obtained were organized and analyzed with IBM SPSS statistical software (version 22.0).

## Results

Table 1 describes the crutch loads of the first 4 walks (1 without feedback, 2-4 with feedback). Loads are displayed as a percentage of the ideal load requested (50% of each subject’s body weight), with the optimum load subsequently set to 100%. The ideal load permissible tolerance was ±10% (ie, ±5%, or a load of 95% to 105%). See Multimedia Appendix 3 for a more general visual presentation of these data.

A total of 96/100 errors were observed in the first walk (walk 0, no feedback), most of which involved crutch overloading by 7/10 subjects (subjects 1, 2, 3, 4, 5, 7, and 9). Errors ranged from 61.09% (subject 8) to 203.98% (subject 1). The loads of subjects 2, 6, and 9 were particularly nonhomogeneous.

The first walk with double-bar feedback (walk 1) began with errors similar to the no-feedback walk errors, generally followed by attempts at correction. Therefore, progress toward the ideal load and fluctuations above and below the optimal load were observed until the optimal load was achieved and maintained as much as possible in the successive walks.

**Table 1:** Percentage of the loads applied on the crutches during the first 4 walks, with an optimum load of 100% and permitted error range of 95%-105%.

Feedback with crutch supports			Subject 1		Subject 2		Subject 3		Subject 4		Subject 5		Subject 6		Subject 7		Subject 8		Subject 9		Subject 10
**Walk 0: Computer feedback^a^**																					
	1	201.08-O^b^		133.98-O		146.39-O		121.07-O		107.98-O		90.41-U^c^		149.87-O		70.70-U		143.20-O		81.67-U	
	2	198.19-O		130.93-O		144.68-O		125.52-O		109.89-O		86.64-U		153.81-O		72.87-U		157.48-O		78.40-U	
	3	199.28-O		137.59-O		143.45-O		126.71-O		109.13-O		88.70-U		153.28-O		72.56-U		169.39-O		77.31-U	
	4	202.17-O		108.46-O		142.47-O		123.44-O		106.84-O		91.44-U		153.28-O		68.53-U		167.01-O		81.31-U	
	5	203.98-O		121.78-O		143.70-O		122.55-O		107.60-O		92.81-U		147.77-O		71.01-U		148.64-O		82.76-U	
	6	201.45-O		120.39-O		145.41-O		126.11-O		*103.04^d^*		*95.21*		154.59-O		66.36-U		158.16-O		78.40-U	
	7	198.55-O		122.05-O		145.17-O		123.74-O		*104.56*		94.52-U		150.66-O		67.91-U		166.67-O		79.49-U	
	8	201.81-O		131.21-O		143.70-O		122.55-O		109.89-O		86.99-U		147.51-O		62.33-U		171.43-O		76.23-U	
	9	203.25-O		128.99-O		142.23-O		126.41-O		*104.94*		87.67-U		155.12-O		61.09-U		172.11-O		81.31-U	
	10	195.66-O		110.68-O		145.90-O		120.18-O		107.22-O		82.53-U		148.03-O		80.62-U		164.97-O		86.03-U	
**Walk 1 (double bar): Screen feedback^e^**																					
	1	150.09-O		144.80-O		158.14-O		123.44-O		117.11-O		83.22-U		162.47-O		66.98-U		148.30-O		78.40-U	
	2	138.88-O		154.79-O		154.47-O		122.26-O		112.93-O		88.70-U		144.88-O		88.06-U		191.84-O		91.83-U	
	3	118.63-O		128.16-O		80.29-U		*97.63*		*96.20*		91.78-U		122.57-O		109.77-O		143.20-O		*101.27*	
	4	*101.99*		134.26-O		131.95-O		108.01-O		*98.48*		*96.23*		109.71-O		*102.02*		94.90-U		*102.00*	
	5	109.95-O		82.39-U		124.11-O		72.11-U		*97.72*		94.52-U		*101.05*		*104.19*		72.45-U		*101.63*	
	6	92.22-U		85.71-U		85.92-U		76.85-U		*97.34*		*97.95*		*100.52*		*102.95*		87.76-U		*99.46*	
	7	*98.01*		93.20-U		*95.96*		82.79-U		*100.38*		*100.00*		*100.79*		*100.47*		*104.42*		*101.27*	
	8	*103.44*		*97.36*		*99.14*		93.77-U		*99.24*		90.75-U		*97.38*		107.60-O		153.74-O		*95.83*	
	9	*99.46*		*101.25*		*104.53*		*96.44*		*98.86*		94.52-U		*102.10*		106.05-O		*104.08*		94.74-U	
	10	*98.73*		110.40-O		*99.39*		*96.14*		*98.10*		*97.95*		*101.57*		*100.47*		120.41-O		*100.91*	
**Walk 2 (double bar): screen feedback^e^**																					
	1	129.48-O		117.61-O		106.49-O		84.87-U		*100.00*		106.85-O		109.45-O		110.70-O		142.18-O		*103.09*	
	2	111.75-O		106.52-O		*104.53*		93.47-U		*101.90*		*103.77*		106.56-O		88.99-U		131.29-O		*104.17*	
	3	*100.18*		*97.64*		*99.39*		108.90-O		*100.38*		*101.37*		*102.89*		91.78-U		*96.60*		107.80-O	
	4	*95.84*		*100.69*		*99.88*		105.64-O		*101.90*		*100.68*		*104.46*		93.64-U		87.07-U		*102.00*	
	5	*101.99*		107.91-O		*95.96*		108.90-O		*103.04*		108.56-O		*103.94*		*97.98*		*97.62*		*101.27*	
	6	*103.80*		*102.08*		*99.14*		*102.67*		*100.38*		*103.42*		*99.21*		111.01-O		119.05-O		*103.81*	
	7	*101.63*		*97.36*		*96.45*		*98.22*		*99.24*		*102.40*		*100.00*		*103.88*		116.33-O		*99.46*	
	8	*100.90*		*98.75*		*95.23*		83.98-U		*98.48*		*101.71*		*101.31*		*101.71*		*104.42*		*98.37*	
	9	*101.27*		*100.14*		92.78-U		*99.70*		*100.00*		*103.08*		*102.10*		*99.53*		*100.68*		*100.54*	
	10	*100.54*		*101.80*		*98.16*		*102.97*		*101.52*		*101.71*		*99.74*		*97.67*		109.86-O		*103.45*	
**Walk 3 (double bar): screen feedback^e^**																					
	1	*99.82*		107.35-O		*97.43*		91.69-U		*98.48*		*101.37*		*99.21*		109.15-O		144.90-O		*99.09*	
	2	91.86-U		*104.85*		*97.43*		*96.74*		*100.00*		*103.42*		*98.69*		*104.81*		130.61-O		*97.28*	
	3	*98.01*		*102.91*		*99.39*		*99.11*		*98.86*		105.14-O		*100.79*		*101.09*		*97.28*		*99.82*	
	4	*102.35*		*104.02*		*101.10*		*102.67*		*98.10*		*99.66*		*102.10*		*100.47*		86.05-U		*103.81*	
	5	*104.52*		*103.19*		*102.57*		*101.19*		*98.48*		*99.32*		*98.16*		*102.64*		*101.36*		*102.72*	
	6	105.24-O		*102.36*		*97.43*		*99.41*		*100.76*		*97.95*		*103.15*		*101.71*		*96.60*		*98.00*	
	7	*102.71*		*104.30*		*99.39*		*98.52*		*103.04*		*98.63*		*104.20*		*99.53*		*100.68*		*97.28*	
	8	*103.44*		105.41-O		*97.18*		*103.26*		*100.38*		*101.71*		*102.62*		*103.88*		111.22-O		*98.37*	
	9	*103.44*		107.91-O		*99.63*		*100.59*		*98.86*		*100.00*		*101.31*		*101.09*		115.31-O		*100.18*	
	10	*100.18*		105.13-O		*99.39*		*101.48*		*98.10*		*104.79*		*102.89*		*99.22*		*103.74*		*103.45*	

^a^Only for physiotherapist/researcher.

^b^O: overloaded.

^c^U: underloaded.

^d^Correctly applied loads are in italics.

^e^For patients.

**Figure 8:**
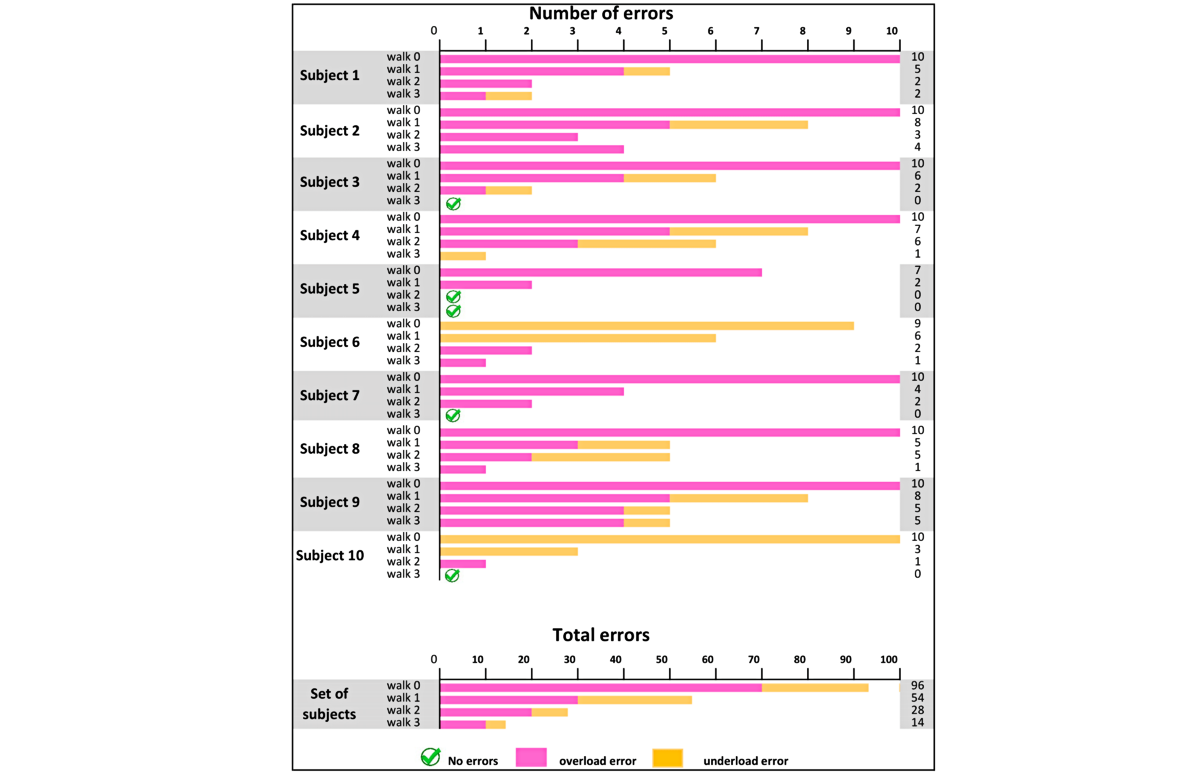
Graphical representation of the number of errors in the weight supported by the crutches on each walk and per subject.

Figure 8 presents the number of errors in the loads applied on the crutches in the first 4 walks, distinguishing between underload errors, overload errors, and the total for each subject and walk. The numerical data are shown in Multimedia Appendix 4.

**Figure 9:**
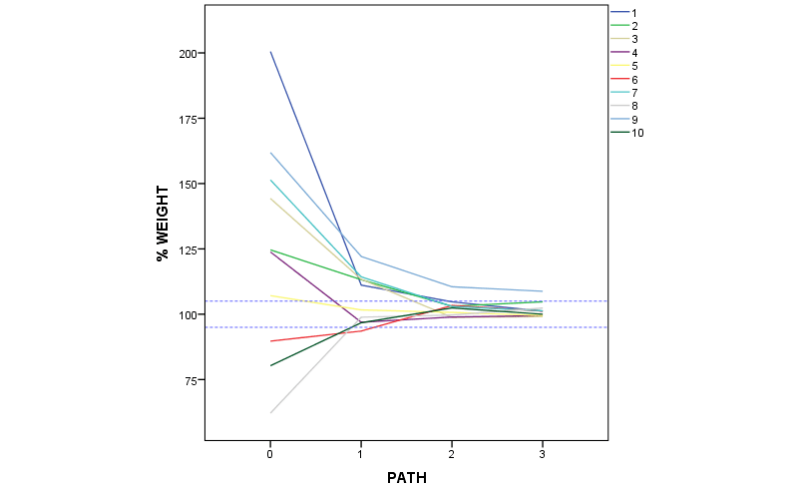
Line graph representing the mean percentage loads (y-axis) for each subject (different colors) in the 4 walks (x-axis).

Table 2 and Figure 9 show the mean percentage loads exerted on the crutches in each walk. The optimal load is considered to be 100%, and the error tolerance range (10%) was between 95% and 105%. The mean error table (Table 2) would be meaningless without Table 1 and Figure 8. For example, if a subject makes extreme overload and underload errors, the mean might seem to be within the tolerated range and therefore correct, but it is not. However, if all errors are overload or underload errors, or the loads are homogeneous, the clinical interpretation of the results in Table 2 would be different.

The Wilcoxon matched-pairs signed-rank test, which was used to evaluate each subject’s progress, showed significant differences in the reduction of overload errors when comparing the baseline values to each of their feedback walks: walk 0 vs walk 1 (*P*=.02), walk 0 vs walk 2 (*P*=.03), walk 0 vs walk (*P*=.02). However, there were no significant differences in the values of the underload errors. The practice walks of different subjects were not compared with each other. A confidence level of 95% was considered, and the experimental *P* value was evaluated at a significance level of 5%. The table with all *P* values is provided in Multimedia Appendix 5.

**Table 2:** Mean percentage (range) loads applied on the crutches during the first 4 walks, with an optimal load of 100% and a permitted error range of 95%-105%.

Subject	Walk 0	Walk 1	Walk 2	Walk 3
1	200.54 (195.66-203.98)-U^a^	111.14 (92.22-150.09)-U	*104.74 (95.84-129.48)^b^*	*101.16 (91.86-105.24)*
2	124.61 (108.46-137.59)-U	113.23 (82.39-154.79)-U	*103.05 (97.36-117.61)*	*104.74 (102.36-107.91)*
3	144.31 (142.23-146.39)-U	113.39 (80.29-158.14)-U	*98.80 (92.78-106.49)*	*99.09 (97.18-102.57)*
4	123.83 (120.18-126.71)-O^c^	*96.94 (72.11-123.44)*	*98.93 (83.98-108.90)*	*99.47 (91.69-103.26)*
5	107.11 (103.04-109.89)-O	*101.64 (96.20-117.11)*	*100.68 (98.48-103.04)*	*99.51 (98.10-103.04)*
6	89.69 (82.53-95.21)-O	93.56 (83.22-100.00)-O	*103.35 (100.68-108.56)*	*101.20 (97.95-105.14)*
7	151.39 (147.51-155.12)-U	114.30 (97.38-162.47)-U	*102.97 (99.21-109.45)*	*101.31 (98.16-104.20)*
8	62.14 (61.09-72.87)-O	*98.86 (66.98-109.77)*	*99.69 (88.99-111.01)*	*102.36 (99.22-109.15)*
9	161.91 (143.20-172.11)-U	122.11 (72.45-191.84)-U	110.51 (87.07-142.18)-U	108.78 (86.05-144.90)-U
10	80.29 (76.23-86.03)-O	*96.73 (78.40-102.00)*	*102.40 (98.37-107.80)*	*100.00 (97.28-103.81)*

^a^U: underload mean error.

^b^Correctly applied mean loads are in italics.

^c^O: overload mean error.

The results regarding the right and left crutch load imbalance errors made during the three double-bar walks were as follows: 0 errors for subjects 1, 3, 5, 6, 7, and 10; 1 error for subjects 4 (walk 1), 8 (walk 2), and 9 (walk 1); and 6 fluctuation errors for subject 2 (3 errors in walk 1, 2 errors in walk 2, and 1 error in walk 3).

Subjects 2 and 9 performed extra walks with other types of feedback, namely balance bar and single bar. The research physiotherapist made this decision after noting that these subjects were finding it difficult to achieve their objectives and needed to focus attention on a specific aspect.

Subject 9 performed 2 more walks with the single-bar feedback to focus on the total load, and progressed from 8, 5, and 5 errors in the three double-bar walks (Figure 8 and Multimedia Appendix 4), respectively, to 3 (first single-bar walk) and 1 (second single-bar walk) errors, thereby completing the test.

Subject 2 performed 2 more walks with the balance-bar feedback to focus on the left and right crutch load balance, and progressed from 3, 2, and 1 imbalances with load fluctuations in the three double-bar walks, respectively, to 2 imbalances with fluctuations (first balance-bar walk) and 0 without fluctuations (second balance-bar walk), thereby completing the test.

## Discussion

### Principal Results

The innovative multifunctional desktop program GCH Control Software 1.0 monitors crutch loads, which can be controlled by real-time graphic and numerical displays during assisted gait practice. The software contains a versatile feedback mechanism correcting any wrongly applied loads, ensuring greater clinical accuracy. The integrated database can be used not only to objectively evaluate the progress in loads of the subjects but also to comparatively analyze the system’s efficiency and draw up clinical action protocols. Moreover, we performed a pilot implementation trial to assess the functionality of the feedback mechanism.

The application environment is basically associated with orthopedics and traumatology [6,38,41,45], and particularly to temporary processes of functional gait recovery of patients with lower limb injuries or surgeries [45] such as ligamentoplasty of the anterior cruciate ligament of the knee [36], a knee or hip prosthesis [21], or a knee osteotomy [40]. In all of these cases, treatment of the lesion starts with no load on the injured limb, which is followed as recovery progresses by partial loads that gradually increase up to a total body weight load. The GCH System is also useful in the fields of gerontology [31,32], neurology [46], pediatrics [46,47], or rheumatology [33], as mentioned earlier. If the applications are extended to these fields where the lesions tend to be chronic, orthotic devices must also be maintained over time [48], increasing the opportunities of using the method. New research prospects providing innovative clinical action protocols for users with disabilities or even normal age deterioration are therefore possible.

This study, like many other research projects [4,15,23,43,49-51], advocates technology and information technology effectiveness in functional recovery processes in general, and specifically in gait. However, effectiveness does not only refer to the intervention itself, in this case based on technological feedback, but also extends to the (global or analytical) initial, continuous, and final assessments of each essential gait parameter [52]. GCH Control Software evaluates the maximum force applied to the crutches in each support and therefore indirectly the unloading of the affected lower limb, which is one of the essential parameters [1,39] in assisted gait with two-stage partial unloading. This peak clinical moment matches the midstance phase of the affected limb (ie, where the limb remains upright and with single stance or without the help of the other limb in the air; midswing phase of the contralateral limb) [53,54]. Unloading relies exclusively on the crutches. The software also monitors if the assistive devices touch the ground at the same time and if their loads are balanced and increase progressively without fluctuations. These data are related to other essential parameters of two-stage gait with forearm crutches, as shown by the CHAGS [1] scale of observational aided gait assessment. Three of its ten load-independent items are directly related to this information: “simultaneous support of both crutches and foot,” “step rhythm” (all steps have to be carried out in the same time), and “fluency” (assisted gait is performed automatically, decisively, and without hesitation).

The consequence drawn by the authors is that mutually complementary and objective technological and observational measurement tools need to be applied [55]. Similarly, despite a major goal of technology system-based clinical interventions to offer patients autonomy, they must also rely on the human eye, hands, intuition, and professional decisions [20]. In the specific case of the GCH Control Software feedback mechanism, the physiotherapist’s presence is shown to optimize the technology features.

### Versatile Feedback

GCH Control Software can be adapted very easily to each patient’s specific characteristics, regardless of their main disease, such as their level of coordination skills [56], previous experience [56] in handling forearm crutches, age, and visual or acoustic disorders. This is because the software provides a wide range of extrinsic feedback, ensuring individualized interventions for achieving specific or general therapeutic goals when recovering gait functions. This feedback can be provided to the clinical professional, the user, or both at the same time, although the clinical professional, usually a physiotherapist, receives more detailed and extensive information than the user who is performing a dual task [57], and should only be given very specific feedback in small amounts for the intervention to be effective.

As for the feedback modalities considered (ie, acoustic, visual, and acoustic-visual), patients receive two kinds of acoustic feedback: computer-based verbal feedback from the physiotherapist and the beeps generated by the software. The advantage of the verbal feedback [49] is that the physiotherapist can decide what information to give in line with the patient’s needs. This is particularly useful if the subject has poor coordination skills, as noted in the dual tasks [49]. Subjects can be given only information about their results (KR feedback) or also about their performance (KP feedback), and can receive information that only describes a mistake (descriptive feedback) or that suggests the strategy for correcting it (prescriptive feedback). The clinician can also choose between giving the information while the patient is walking (concurrent feedback) or afterward (terminal feedback), and among immediate terminal feedback, delayed terminal feedback, or summary terminal feedback (after several walks). The beeps are immediate terminal feedback, because subjects receive the signal immediately when they begin to take a step using support for the affected limb with crutches. The acoustic signal tells subjects if they have reached the correct maximum load at the end of each support, which is why it is given immediately. However, during free cadence walking [58], steps are taken very quickly, and the period of time between the maximum load and end of the support is too imperceptible so that the signal can be considered a real-time signal, and thus it can be interpreted clinically as concurrent feedback. This is particularly true if the gait is regarded as a cyclical process [59] and each crutch support or step is not assessed independently.

With regard to visual information, the main advantage is its continuity (continuous concurrent feedback) by showing how the loads applied on the crutches steadily increase and decrease in double-bar, single-bar, and balance-bar feedback. Moreover, the double-bar and single-bar feedback show if each crutch support is correct or incorrect (immediate terminal feedback).

The software’s multiple independent biofeedback options can be extended by using various combinations of these options. Numerous studies have assessed the effectiveness of various types of feedback [26,27,49,60,61] and their combinations. A recent systematic review [43] of technology-based feedback and its efficacy in improving gait parameters concluded that immediate visual feedback was the most widely used, followed by terminal and immediate acoustic feedback.

### GCH Pilot Implementation

In line with several previous studies [11,23,27,51], the results obtained in this study suggest that feedback mechanism–based interventions are satisfactory. The pilot experiment showed that subjects applied significantly more accurate loads on the forearm crutches after only a few minutes, or even a few seconds in some cases. These preliminary data should be confirmed in subsequent longer studies with larger samples. Nevertheless, these findings suggest that interventions are more effective, and even more efficient, owing to the feedback immediacy. Although the participant inclusion criteria were favorable to achieve positive results (ie, all participants had to have experience in two-stage bilateral assisted walking with partial unloading, and their technical gesture had to be acceptable based on a score of 3-4 points for each CHAGS item [1]), only one participant (Subject 5) had previous experience of load measurements, and with the same software.

The crutch load measurements without feedback (ie, walk 0) showed that all subjects made significant overload and underload mistakes (even with twice the requested load). Most of the subjects (7/10) overloaded the crutches, thereby unloading their body weight more than necessary, with associated clinical disadvantages. For example, in older patients with osteoporosis [32,42], unnecessary unloading is not appropriate because it inhibits osteoblastic action and increases osteoclastic action. Even if there is no underlying disease, the tendency is always to use the largest possible weight-bearing load [42], without damaging the injury, to stimulate circulation and muscle tone, and therefore optimize the patient’s overall functional recovery. This is why frequent two-stage assisted walking without the injured lower limb touching the ground (foot in the air) is not recommended, unless necessary. The negative clinical consequences of underloading the crutches can be even more severe with certain injuries or types of surgery such as noncemented knee or hip prostheses, osteotomy [40,41], or autologous chondrocyte implantation [62]. In such cases, overloading the injured area can lead to further surgery, longer functional recovery times, or other side effects or sequelae, among other drawbacks [42]. Several subjects (3/10), despite being coordinated and having used crutches previously, made these mistakes constantly without feedback. The case of Subject 10 was particularly interesting, whose technical gesture with homogeneous loads were perfect, and remained well below the requested load on the crutches without feedback. This is a clear example of the usefulness of GCH Control Software, even with skilled and experienced patients.

In the three double-bar feedback walks, maximum crutch load accuracy errors decreased in all participants from the first to the third walk. At the start of the tests, and especially the first test, participants made more mistakes and differences between the requested and applied force were larger. Shortly afterward, the visual and acoustic information provided by the software helped them to adjust the loads. The biggest errors were made during the first walk; however, 3 subjects did not make any mistake in the third walk, and another 3 subjects only made one mistake, representing a minimum difference. One subject did not make any mistakes in the second or third walk. However, two of the subjects did not achieve the expected results in the double-bar walk.

Subject 2 began with very high loads and made 8/10 mistakes in the first walk, which decreased, but to a lesser extent than observed for the majority of participants (ie, 3 errors made in the second walk and 4 errors made in the third walk). During the 3 walks, it was observed that the crutches did not always load evenly, and the right and left bars fluctuated, even though the final weight of each crutch met the requirements (6/30 crutch balance errors). The physiotherapist decided to continue the intervention and apply the third type of feedback (balance bar) to allow the subject to focus their attention on leaning on both crutches at the same time and ensure even crutch loads, regardless of the total amount. As expected, the subject’s performance improved remarkably with only one walk, after scoring 3/4 points in the CHAGS scale item on simultaneously using both crutches and the affected limb [1]. Therefore, occasionally, the subject did not meet this goal. The GCH Control Software feedback seems to not only facilitate load monitoring and accuracy but can also detect and correct other erroneous parameters that are equally essential in crutch-assisted gait (eg, leaning on both crutches simultaneously as in the case of Subject 2). This subject is a clear example that technological assessment and feedback (GCH System linked with GCH Control Software), combined with observational assessment and direct feedback from the physiotherapist (CHAGS score), can be used together and in a complementary manner, thereby individualizing each intervention [1].

The number of errors made by Subject 9 dropped in the double-bar feedback walks (from 7 to 5); however, there were still numerous disparate inaccuracies, in line with the prefeedback walk. Nevertheless, the subject maintained the crutch load balance. After analyzing these data, the physiotherapist asked the subject to continue the intervention with the single-bar display, and the subject made only 3 mistakes in the first walk and 1 mistake in the second walk. This improvement was explained by having focused the subject’s attention [56] on the total load without displaying independent data from each crutch, which were not necessary in this case. The subject also walked more times and therefore spent more time practicing, which must also be taken into account for interpreting this improvement [56].

At the end of the pilot experiment, the subjects commented that they had been surprised by the difference between how they actually performed and how they thought they had performed. They found the test to be easy, comfortable, and effective. Even those who needed a few extra screens spoke positively about GCH Control Software and GCH System in general. They also stated that despite hearing the acoustic signals, they were generally more aware of the visual signals.

### Limitations and Prospects

This study was limited to an extent by the sample size and short duration of the intervention. Longer prospective clinical trials should be performed, with large samples and control groups to ascertain the effectiveness and efficiency of the different types of graphical biofeedback (double bar, single bar, and balance), acoustic feedback (verbal and/or beep), or combined feedback available with GCH Control Software. For example, acoustic feedback using beeps and visual feedback could be applied separately, followed by evaluation of whether (and in which cases) acoustic feedback alone suffices in the long term. As explained earlier, the more options there are, the easier it is to customize or adapt the system to each patient during the treatment, both in line with individual characteristics (eg, coordination) and specific temporary musculoskeletal injuries or chronic situations due to illness or age. Clinical trials are therefore required to address different pathologies and contexts in the various branches of medicine to develop effective clinical action protocols.

Given the rapid progress of information technology, future adaptations of GCH Control Software to new communication systems will be necessary. However, these adaptations would be implemented at the lower levels of the software stack. The way feedback information is presented to patients and physiotherapists would not be affected by these adaptations.

### Conclusions

This study describes GCH Control Software, the multifunctional desktop software associated with GCH System 2.0, which measures loads exerted on forearm crutches by patients who need to partially unload a pathological lower limb.

The program graphically and numerically monitors loads in real time during assisted gait interventions, and uses a versatile, efficient feedback mechanism to correct the accuracy of wrongly applied loads. It also shows if subjects use both crutches at the same time in bilateral walking and how evenly they distribute the load between them. The program features a patient and test database that can be used to objectify applied load progress and comparatively analyze the system’s effectiveness in different clinical contexts, enabling drawing up clinical action protocols based on scientific evidence.

Satisfactory findings were obtained in the complementary pilot implementation tests of the feedback mechanism functionality. Although an ideal sample was selected, all subjects made errors exerting loads on the forearm crutches during bilateral gait in the baseline test, indicating the necessity or appropriateness of the method presented. They achieved greater accuracy with only one brief session. Although one subject found it difficult to balance the loads between the two crutches and fluidly apply the loads, these issues were ultimately resolved with feedback. However, any clinical data provided herein should be interpreted with caution. Future research should focus on how participants’ accuracy changes over several weeks to confirm the findings.

## Appendix

Multimedia Appendix 1 Crutch step count, displaying the total number of supports, number of correct supports, and number of wrong supports. Each crutch support is also represented numerically, showing the load applied to the left, right, and both crutches, and whether or not the support was correct.

Multimedia Appendix 2 Subject demographics.

Multimedia Appendix 3 Visual representation of correct (green), overloaded (pink), and underloaded (gold) loads applied on the crutches during the first 4 walks (related to Table 1).

Multimedia Appendix 4 Number of errors in the weight supported by the crutches on each walk and per subject.

Multimedia Appendix 5 <italic>P</italic> values from the Wilcoxon matched-pairs signed-rank test.

## Abbreviations

CHAGS: Chamorro Assisted Gait Scale
KP: knowledge of performance
KR: knowledge of results
