# Supplementary figures and images for: Versatile GCH Control Software for Correction of Loads Applied to Forearm Crutches During Gait Recovery Through Technological Feedback: Development and Implementation Study
Source: J Med Internet Res. 2021 Sep 22;23(9):e27602. doi: 10.2196/27602 (PMC8495581; doi:10.2196/27602)

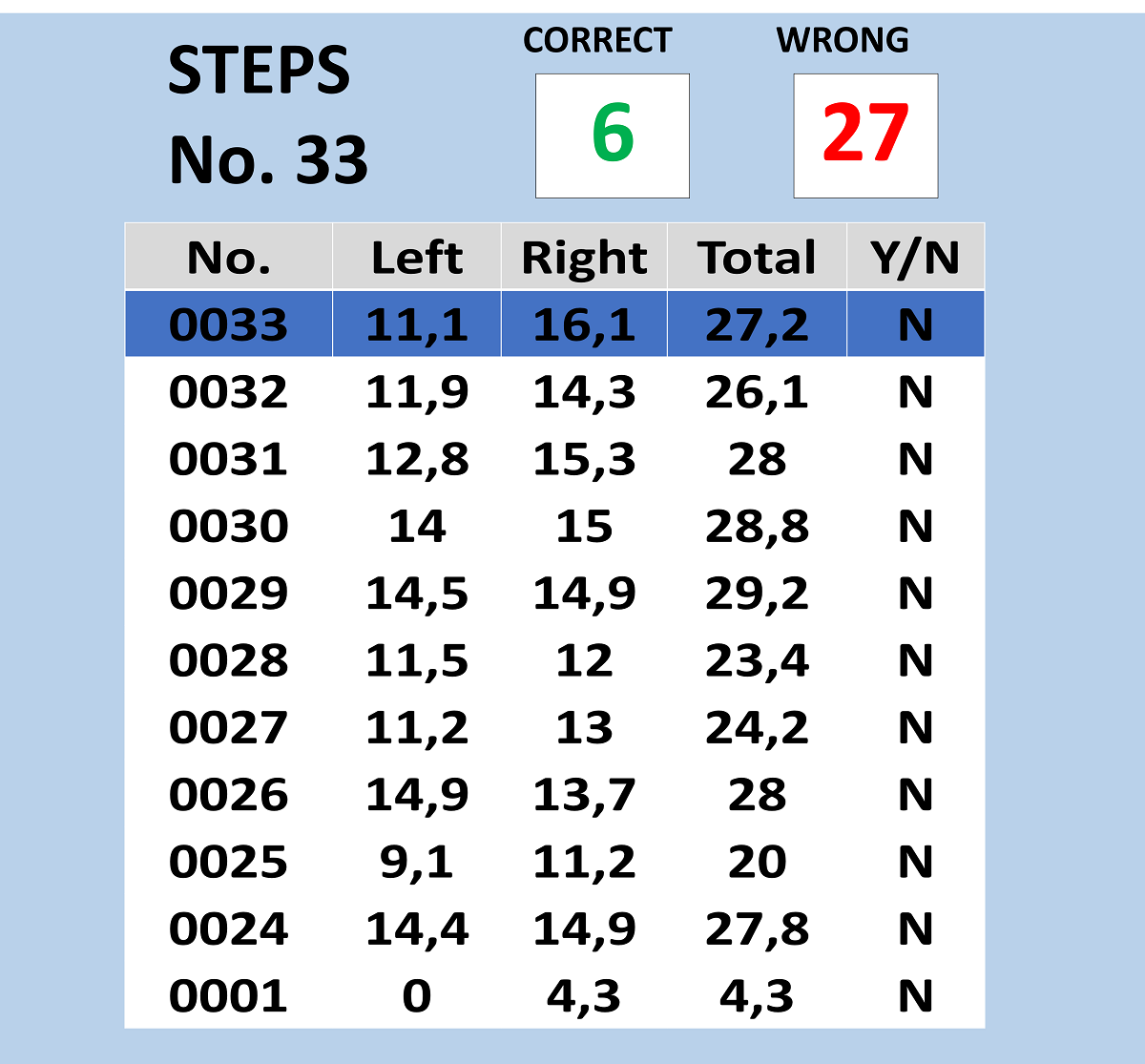

Supplement: Multimedia Appendix 1 [file jmir_v23i9e27602_app1.png]
