# Supplementary material for: Versatile GCH Control Software for Correction of Loads Applied to Forearm Crutches During Gait Recovery Through Technological Feedback: Development and Implementation Study
Source: J Med Internet Res. 2021 Sep 22;23(9):e27602. doi: 10.2196/27602 (PMC8495581; doi:10.2196/27602)
Supplement: Multimedia Appendix 2 [file jmir_v23i9e27602_app2.docx]

**Appendix 2.** Subject demographics.

| ***Subject*** | ***Age*** | ***Gender*** | ***Weight (kg)*** | ***Height (cm)*** | ***bmi*** ^a^ |
| --- | --- | --- | --- | --- | --- |
| ***1*** | 26 | F | 55.3 | 164 | 20.6 |
| ***2*** | 31 | M | 72.1 | 175 | 23.5 |
| ***3*** | 23 | M | 81.7 | 184 | 24.1 |
| ***4*** | 55 | F | 67.4 | 171 | 23 |
| ***5*** | 45 | F | 52.6 | 159 | 20.8 |
| ***6*** | 42 | M | 58.4 | 168 | 20.7 |
| ***7*** | 21 | M | 76.2 | 178 | 24 |
| ***8*** | 44 | M | 64.5 | 173 | 21.6 |
| ***9*** | 51 | F | 58.8 | 166 | 21.3 |
| ***10*** | 36 | F | 55.1 | 158 | 22.1 |
|  |  |  |  |  |  |

^a^ BMI; body mass index= weight [kg]/ height [m2]. Normal values: 18.5-24.9 (National Heart, Lung, and Blood Institute, USA).
