# Supplementary material for: Versatile GCH Control Software for Correction of Loads Applied to Forearm Crutches During Gait Recovery Through Technological Feedback: Development and Implementation Study
Source: J Med Internet Res. 2021 Sep 22;23(9):e27602. doi: 10.2196/27602 (PMC8495581; doi:10.2196/27602)
Supplement: Multimedia Appendix 3 [file jmir_v23i9e27602_app3.docx]

**Multimedia Appendix 3**. Visual representation of correct (green), overloaded (pink), and underloaded (gold) loads applied on the crutches during the first 4 walks (related to Table 1).

| **PERMISSIBLE WEIGHT-BEARING ERROR 10% (95%-105%)** | **CRUTCH SUPPORTS** | **SUBJECTS** | | | | | | | | | |
| --- | --- | --- | --- | --- | --- | --- | --- | --- | --- | --- | --- |
|  |  | **1** | **2** | **3** | **4** | **5** | **6** | **7** | **8** | **9** | **10** |
| **WALK 0 -COMPUTER FEEDBACK**  **(only for physiotherapist/**  **researcher)-** | WALK 0 (1) |  |  |  |  |  |  |  |  |  |  |
|  | WALK 0 (2) |  |  |  |  |  |  |  |  |  |  |
|  | WALK 0 (3) |  |  |  |  |  |  |  |  |  |  |
|  | WALK 0 (4) |  |  |  |  |  |  |  |  |  |  |
|  | WALK 0 (5) |  |  |  |  |  |  |  |  |  |  |
|  | WALK 0 (6) |  |  |  |  |  |  |  |  |  |  |
|  | WALK 0 (7) |  |  |  |  |  |  |  |  |  |  |
|  | WALK 0 (8) |  |  |  |  |  |  |  |  |  |  |
|  | WALK 0 (9) |  |  |  |  |  |  |  |  |  |  |
|  | WALK 0 (10) |  |  |  |  |  |  |  |  |  |  |
| **DOUBLE BAR: 1^st^ WALK -SCREEN FEEDBACK**  **(for patients)-** | WALK 1 (1) |  |  |  |  |  |  |  |  |  |  |
|  | WALK 1 (2) |  |  |  |  |  |  |  |  |  |  |
|  | WALK 1 (3) |  |  |  |  |  |  |  |  |  |  |
|  | WALK 1 (4) |  |  |  |  |  |  |  |  |  |  |
|  | WALK 1 (5) |  |  |  |  |  |  |  |  |  |  |
|  | WALK 1 (6) |  |  |  |  |  |  |  |  |  |  |
|  | WALK 1 (7) |  |  |  |  |  |  |  |  |  |  |
|  | WALK 1 (8) |  |  |  |  |  |  |  |  |  |  |
|  | WALK 1 (9) |  |  |  |  |  |  |  |  |  |  |
|  | WALK 1 (10) |  |  |  |  |  |  |  |  |  |  |
| **DOUBLE BAR:**  **2^nd^ WALK -SCREEN FEEDBACK**  **(for patients)-** | WALK 2 (1) |  |  |  |  |  |  |  |  |  |  |
|  | WALK 2 (2) |  |  |  |  |  |  |  |  |  |  |
|  | WALK 2 (3) |  |  |  |  |  |  |  |  |  |  |
|  | WALK 2 (4) |  |  |  |  |  |  |  |  |  |  |
|  | WALK 2 (5) |  |  |  |  |  |  |  |  |  |  |
|  | WALK 2 (6) |  |  |  |  |  |  |  |  |  |  |
|  | WALK 2 (7) |  |  |  |  |  |  |  |  |  |  |
|  | WALK 2 (8) |  |  |  |  |  |  |  |  |  |  |
|  | WALK 2 (9) |  |  |  |  |  |  |  |  |  |  |
|  | WALK 2 (10) |  |  |  |  |  |  |  |  |  |  |
| **DOUBLE BAR:**  **3^rd^ WALK -SCREEN FEEDBACK**  **(for patients)-** | WALK 3 (1) |  |  |  |  |  |  |  |  |  |  |
|  | WALK 3 (2) |  |  |  |  |  |  |  |  |  |  |
|  | WALK 3 (3) |  |  |  |  |  |  |  |  |  |  |
|  | WALK 3 (4) |  |  |  |  |  |  |  |  |  |  |
|  | WALK 3 (5) |  |  |  |  |  |  |  |  |  |  |
|  | WALK 3 (6) |  |  |  |  |  |  |  |  |  |  |
|  | WALK 3 (7) |  |  |  |  |  |  |  |  |  |  |
|  | WALK 3 (8) |  |  |  |  |  |  |  |  |  |  |
|  | WALK 3 (9) |  |  |  |  |  |  |  |  |  |  |
|  | WALK 3 (10) |  |  |  |  |  |  |  |  |  |  |

Green: correctly applied loads; pink: overloaded; gold: underloaded.
