# Supplementary material for: Versatile GCH Control Software for Correction of Loads Applied to Forearm Crutches During Gait Recovery Through Technological Feedback: Development and Implementation Study
Source: J Med Internet Res. 2021 Sep 22;23(9):e27602. doi: 10.2196/27602 (PMC8495581; doi:10.2196/27602)
Supplement: Multimedia Appendix 4 [file jmir_v23i9e27602_app4.docx]

**Multimedia Appendix 4.** Number of errors in the weight supported by the crutches on each walk and per subject.

|  | **Errors - Walk 0** | | | **Errors - Walk 1** | | | **Errors - Walk 2** | | | **Errors - Walk 3** | | |
| --- | --- | --- | --- | --- | --- | --- | --- | --- | --- | --- | --- | --- |
| *Subject* | **Under-load** | **Over-**  **load** | **Total** | **Under-**  **load** | **Over-**  **load** | **Total** | **Under-**  **load** | **Over-**  **load** | **Total** | **Under-**  **load** | **Over-**  **load** | **Total** |
| 1 | 0 | 10 | **10** | 1 | 4 | **5** | 0 | 2 | **2** | 1 | 1 | **2** |
| 2 | 0 | 10 | **10** | 3 | 5 | **8** | 0 | 3 | **3** | 0 | 4 | **4** |
| 3 | 0 | 10 | **10** | 2 | 4 | **6** | 1 | 1 | **2** | 0 | 0 | **0** |
| 4 | 0 | 10 | **10** | 4 | 3 | **7** | 3 | 3 | **6** | 1 | 0 | **1** |
| 5 | 0 | 7 | **7** | 0 | 2 | **2** | 0 | 0 | **0** | 0 | 0 | **0** |
| 6 | 9 | 0 | **9** | 6 | 0 | **6** | 0 | 2 | **2** | 0 | 1 | **1** |
| 7 | 0 | 10 | **10** | 0 | 4 | **4** | 0 | 2 | **2** | 0 | 0 | **0** |
| 8 | 10 | 0 | **10** | 2 | 3 | **5** | 3 | 2 | **5** | 0 | 1 | **1** |
| 9 | 0 | 10 | **10** | 3 | 5 | **8** | 1 | 4 | **5** | 1 | 4 | **5** |
| 10 | 10 | 0 | **10** | 3 | 0 | **3** | 0 | 1 | **1** | 0 | 0 | **0** |
| TOTAL | 29 | 67 | **96** | 24 | 30 | **54** | 8 | 20 | **28** | 3 | 11 | **14** |
