# Supplementary material for: Versatile GCH Control Software for Correction of Loads Applied to Forearm Crutches During Gait Recovery Through Technological Feedback: Development and Implementation Study
Source: J Med Internet Res. 2021 Sep 22;23(9):e27602. doi: 10.2196/27602 (PMC8495581; doi:10.2196/27602)
Supplement: Multimedia Appendix 5 [file jmir_v23i9e27602_app5.docx]

**Multimedia Appendix 5.** *P* values from the Wilcoxon matched-pairs signed-rank test**.**

| ***P*-values** | **walk 0 vs walk 1** | **walk 0 vs walk 2** | **walk 0 vs walk 3** |
| --- | --- | --- | --- |
| **Underload errors** | .89 | .34 | .34 |
| **Overload errors** | .02 | .03 | .02 |
| **Total errors** | .00 | .00 | .00 |
